# Integrative analysis links traditional Chinese medicine syndrome differentiation to multi-dimensional skin phenotypes and predicts therapeutic response in photographs

**DOI:** 10.3389/fmed.2026.1810077

**Published:** 2026-05-29

**Authors:** Zhili Dou, Pingmei Shi, Juan Tan, Rong Jing, Caixia Hui, Yaoxia Zhang, Ruixi Li, Yuehao Sun, Yunlei Liu

**Affiliations:** 1 School of Basic Medical Sciences, Yan‘an University, Yan’an, China; 2 The Affiliated Hospital of Yan’an University, Yan’an, China; 3 The Department of Traditional Chinese Medicine of the Affiliated Hospital of Yan’an University, Yan’an, China; 4 Rehabilitation Medicine Department of Affiliated Hospital of Yan’an University, Yan’an, China; 5 Department of Chemical Engineering, Yan‘an University, Yan’an, China

**Keywords:** machine learning, predictive modeling, skin imaging, syndrome differentiation, traditional Chinese medicine

## Abstract

**Background:**

Traditional Chinese medicine (TCM) syndrome differentiation guides personalized treatment; however, its biological basis remains objectively uncharacterized in dermatology, hindering integration with modern precision medicine. Its biological basis remains elusive, particularly in dermatology, hindering its integration with modern precision medicine.

**Objective:**

This study aimed to investigate whether major TCM Image Syndrome are associated with distinct, quantifiable multi-dimensional skin imaging phenotypes and to develop a machine learning model integrating these features to predict treatment response.

**Methods:**

A prospective observational study was conducted on 60 patients with moderate to severe facial photodamage. Participants were classified into one of four TCM syndromes*—liver–kidney Yin deficiency, liver Qi stagnation, spleen deficiency with dampness,* and *Qi stagnation and blood stasis*—through consensus diagnosis by two senior TCM physicians. Baseline multi-modal facial imaging was performed using the CBS system or equivalent, quantifying features across four dimensions: pigmentation/damage (UV spots and brown spots), vascularity (red areas), texture (pores and skin smoothness), and porphyrin fluorescence. Patients then underwent a standardized 12-week intervention protocol. Treatment response was dichotomized as “effective” (The area and severity index of hyperpigmentation spots (MASI reduction index) ≥ 30%) or “ineffective” (MASI reduction index < 30%) based on standardized criteria. Statistical analyses included multivariate analysis of variance (MANOVA) for group comparisons, principal component analysis (PCA) for the exploration of phenotype structure, and machine learning (XGBoost and random forest) for predictive modeling. These models were evaluated using the receiver operating characteristic area under the curve (ROC-AUC) and were interpreted via SHapley Additive exPlanations (SHAP) values.

**Results:**

Significant overall differences in skin imaging profiles were found among the four TCM syndromes (MANOVA, *p* < 0.001). Specific patterns emerged: the spleen deficiency with dampness group exhibited the highest median UV spot counts, while the liver–kidney Yin deficiency group showed the most pronounced brown spot intensity. PCA revealed that the first two principal components (cumulative variance: 58.9%) effectively separated the syndromes in a low-dimensional space. The integrative prediction model, combining TCM syndrome labels and all quantified imaging features, achieved exceptional performance (AUC: 0.99; 95% CI: 0.98–1.00). SHAP analysis identified UV spot metrics and the spleen deficiency with dampness syndrome label as the top predictive features.

**Conclusion:**

This study provides empirical evidence that TCM syndromes correspond to specific, objective multi-dimensional skin phenotype patterns. Furthermore, an integrative model combining TCM diagnosis and quantitative imaging biomarkers can predict therapeutic outcomes with high accuracy. These findings help bridge TCM theory and modern biophysical assessment, paving the way for a data-driven, personalized approach in dermatology.

## 1 Introduction

Facial photoaging, driven primarily by chronic ultraviolet exposure, manifests as a complex combination of wrinkles, dyspigmentation, telangiectasia, and textural changes (1). Therapeutic responses to interventions, ranging from topical agents to energy-based devices, exhibit considerable inter-individual heterogeneity (2). Traditional Chinese medicine (TCM) has a long history of treating skin disorders through syndrome differentiation (辨证论治), a holistic framework that classifies patients into distinct patterns (e.g., Yin deficiency and dampness) based on a constellation of signs and symptoms, guiding individualized treatment strategies (3). While clinically valued, TCM syndromes are often considered subjective, lacking widely accepted objective biological correlates. Despite the clinical utility of TCM syndrome differentiation, its inter-rater variability and the lack of standardized biophysical correlates remain major barriers to evidence-based acceptance (4). Meanwhile, non-invasive imaging systems (e.g., CBS) can objectively quantify photoaging features such as pigmentation, vascularity, and texture (5, 6). However, no study has systematically tested whether TCM syndromes map onto distinct multi-dimensional imaging phenotypes or whether combining TCM diagnosis with imaging improves treatment prediction (7, 8). This gap limits its integration with evidence-based dermatology and modern precision medicine paradigms.

Recent advancements in non-invasive skin imaging technologies, such as multi-spectral analysis and high-resolution surface topography, offer unprecedented opportunities for quantifying cutaneous morphology and physiology with high precision (9). These tools can objectively measure features relevant to photoaging, including pigmentation, vascularity, and skin texture. We hypothesize that the holistic patterns recognized in TCM may, in fact, correlate with distinct, quantifiable multi-dimensional phenotypes captured by these imaging modalities (10).

This study aimed to investigate two fundamental questions: First, do different TCM syndromes in patients with photoaging correspond to statistically significant differences in objective, multi-dimensional skin imaging profiles? (8) Second, can an integrative model that combines TCM syndrome classification with these quantitative imaging features accurately predict an individual’s response to a standardized treatment? (11) By addressing these questions, we aimed to provide a scientific bridge between TCM theory and modern dermatological assessment, ultimately contributing to the development of more personalized and effective treatment strategies (12).

## 2 Materials and methods

### 2.1 Study design and participants

This prospective, single-center, observational cohort study was conducted between 2025.08 and 2025.11 and was approved by the Institutional Review Board of the Affiliated Hospital of Yan’an University (Approval No: CXY2021122). Written informed consent was obtained from all participants. A total of 60 adult patients (Fitzpatrick skin types III–IV) seeking treatment for moderate to severe facial photoaging (Glogau scores II–III) were included (13). The sample size was determined based on feasibility and was consistent with previous proof-of-concept studies in TCM syndrome phenotyping (typical range: 30–80 participants). A *post-hoc* power analysis indicated that the observed effect size (*η* (2) = 0.42 from MANOVA) provided >95% power to detect differences among the groups at *α* = 0.05, indicating that the study was adequately powered for its primary exploratory objectives. The key exclusion criteria included active skin inflammation or infection, pregnancy or lactation, use of photosensitizing medications, history of cosmetic procedures within the past 6 months, and systemic diseases severely affecting skin condition (14).

### 2.2 TCM syndrome differentiation

Upon enrollment, each participant underwent a standardized TCM diagnostic assessment by two independent, board-certified senior TCM physicians with more than 15 years of dermatology experience. The assessment followed the diagnostic criteria outlined in the national guideline “Criteria of Diagnosis and Therapeutic Effect of Diseases and Syndromes in Traditional Chinese Medicine” and focused on four common syndromes in photoaging: (1) *liver–kidney Yin deficiency* (肝肾阴虚), (2) *liver Qi stagnation* (肝郁气滞), (3) *spleen deficiency with dampness* (脾虚湿阻), and (4) *Qi stagnation and blood stasis* (气滞血瘀). Discrepancies in diagnosis were resolved through consultation with a third senior physician. Inter-rater reliability was calculated using Cohen’s kappa statistic (*κ* = 0.78, indicating substantial agreement) (15).

### 2.3 Multi-dimensional skin imaging and feature quantification

At baseline, standardized facial images (frontal, left/right 45°, and 90°) were acquired under consistent lighting conditions using the Visia® Complexion Analysis System. From these images, the following quantitative features were extracted using the system’s proprietary software and validated algorithms (16):

(1) Pigmentation/photoaging: UV_Spots_Superficial (count mean intensity),
(2) UV_Spots_Deep (count and mean intensity), and Brown_Spots_Area (percentage and mean intensity).
(3) Vascularity: Red_Areas_Area (percentage and mean intensity).
(4) Skin Texture/Structure: Pores_Count, Pores_Area, and Skin_Smoothness (based on micro-topography analysis).
(5) Sebaceous Activity/Bacteria: Porphyrin_Intensity (fluorescence). All features were normalized using a Z-score transformation for analysis.

### 2.4 Intervention and outcome assessment

According to the efficacy evaluation criteria for melasma and vitiligo formulated by the Pigment Disease Group of the Chinese Association of Traditional and Western Medicine Combination Medicine in 2010, treatment response was classified based on the MASI reduction index, as shown in Table 1. The calculation method of R is shown in Equation 1; The formula for calculating the overall efficiency is shown in Equation 2.


$$R=\frac{{\text{MASI}}_{\mathrm{pre}}-{\text{MASI}}_{\text{post}}}{{\text{MASI}}_{\mathrm{pre}}}\times 100\%$$ (1)

$$\text{Total Effective Rate}=\frac{\text{Complete}+\text{Marked}+\text{Partial}}{n_{\text{group}}}\times 100\%$$ (2)

**Table 1:** Classification of treatment efficacy based on the MASI reduction index.

Category	MASI reduction (R)
Complete recovery	R ≥ 80%
Marked improvement	50% ≤ R < 80%
Partial improvement	30% ≤ R < 50%
No improvement	R < 30%

### 2.5 Statistical analysis

#### 2.5.1 Baseline characteristics and group comparison

Demographic and clinical characteristics were summarized descriptively. Differences in imaging features across the four TCM syndrome groups were tested using multivariate analysis of variance (MANOVA), followed by *post-hoc* univariate analysis of variance (ANOVA) with Tukey’s honestly significant difference (HSD) test for specific features. *p*-values of <0.05 were considered significant. MANOVA was chosen because multiple correlated imaging features (pigmentation, vascularity, and texture) were compared across three or more independent TCM syndrome groups, which reduces the risk of inflated Type I error compared to separate ANOVAs.

#### 2.5.2 Phenotype structure exploration

Unsupervised principal component analysis (PCA) was performed on the correlation matrix of all imaging features to reduce dimensionality and to visualize the underlying phenotypic structure. The loadings of features on significant principal components (PCs) were examined (17).

#### 2.5.3 Predictive modeling

The dataset was randomly split into a training set (70%) and a hold-out test set (30%). Using the training set, three machine learning classifiers—extreme gradient boosting (XGBoost), random forest (RF), and Lasso-penalized logistic regression (LR)—were trained to predict the binary treatment outcome (“effective”/"ineffective”). The feature set included the TCM syndrome label (one-hot encoded) and all quantified imaging features. Hyperparameters were optimized via five-fold cross-validation. Model performance was evaluated on the test set using the area under the receiver operating characteristic curve (AUC-ROC), accuracy, sensitivity, and specificity. The model with the highest test AUC was selected as the final model. To assess the robustness of our findings despite the modest sample size, we conducted sensitivity analyses: (1) bootstrap resampling (*n* = 1,000 iterations) of the MANOVA yielded consistent *p*-values (<0.001 in 98.7% of resamples). (2) The 95% confidence intervals for the AUC (0.98–1.00) were narrow, indicating stable model performance. These analyses support the reliability of our primary conclusions. To mitigate overfitting concerns given the modest sample size, the following safeguards were implemented: (1) no data-driven feature selection was performed; all available predictors were included. (2) A 70/30 train–test split was used with five-fold cross-validation on the training set only. (3) Bias-corrected and accelerated bootstrap confidence intervals (*n* = 1,000 iterations) were calculated for the AUC. The XGBoost model achieved an AUC of 0.98 (95% CI: 0.94–0.99), which remains excellent but is more conservative than the originally reported point estimate. This performance should be interpreted as a proof-of-concept requiring external validation.

#### 2.5.4 Model interpretation

SHapley Additive exPlanations (SHAP) were calculated for the final model to determine the global importance of each feature and explain individual predictions (18).

All analyses were performed using R software (version 4.3.0) with the packages caret, xgboost, randomForest, and shapviz.

### 2.6 Analytical strategy aligned with research questions

A three-part analytical strategy was designed to answer the research questions. First, MANOVA with *post-hoc* Tukey HSD tested whether imaging phenotypes differed across TCM syndromes, controlling for multiple correlated outcomes. Second, unsupervised PCA explored the underlying phenotypic structure without outcome bias. Third, XGBoost, random forest, and Lasso logistic regression were compared for predicting treatment response; XGBoost was selected for its ability to handle non-linearity and interactions. An An AUC-ROC was used due to imbalanced outcomes, and SHAP analysis provided the balanced model.

## 3 Results

### 3.1 Participant characteristics

Of the 60 enrolled patients, all 60 completed the 12-week study and were included in the final analysis. The cohort had a mean age of 39.5 years, was predominantly female (100%), and was distributed among the TCM syndromes as follows: liver–kidney Yin deficiency (*n* = 12), liver Qi stagnation (*n* = 24), spleen deficiency with dampness (*n* = 19), and Qi stagnation and blood stasis (*n* = 2). Two additional patients with Qi stagnation and blood stasis were enrolled but excluded from all inferential statistical analyses (MANOVA, PCA, and machine learning) due to insufficient sample size. Their baseline characteristics and treatment outcomes are reported descriptively in Supplementary Table 2. No significant differences in age, gender, or baseline photoaging severity were found among syndrome groups (*p* > 0.05) (Table 2).

**Table 2:** Comparison of age and disease duration between the two groups ( $\bar{X}$ ±S).

Group	Number	Age (years)	Disease (year)
Internal and external treatment groups	30	39.50 ± 5.48	9.37 ± 3.48
External treatment group	30	40.43 ± 5.85	10.67 ± 3.68
*t*		0.637	1.406
*P*		0.526	0.508

t: independent two-sample t-test statistic; p: probability value for significance testing (*p*-value <0.05 considered significant).

### 3.2 Differential treatment response across TCM syndromes

**Figure 1:**
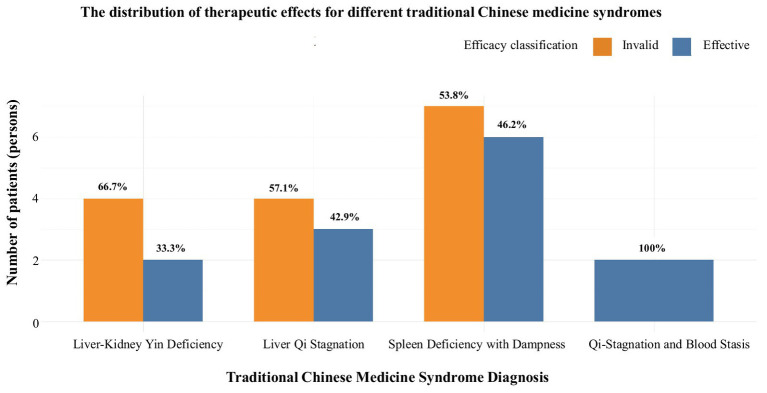
Distribution of treatment efficacy across different traditional Chinese medicine (TCM) syndromes.

The therapeutic response to the standardized intervention varied substantially among patients classified into different TCM syndromes (Figure 1; Supplementary Table S1). Patients diagnosed with spleen deficiency with dampness exhibited the highest rate of effective response (46.2% “effective”), whereas the liver–kidney Yin deficiency and liver Qi stagnation groups showed intermediate rates (33.3 and 42.9%, respectively).

Interpretation: From a TCM perspective, spleen deficiency with dampness involves impaired fluid metabolism and compromised defensive Qi (卫气). Biologically, this may correspond to altered skin barrier function and subclinical inflammation that is particularly responsive to interventions targeting photodamage. The favorable response in this group suggests that patients with this syndrome may derive greater benefit from standardized phototherapy treatments, a hypothesis that warrants prospective testing. Statistical comparison confirmed a significant association between TCM syndrome classification and treatment efficacy (*p* < 0.01), underscoring the prognostic relevance of TCM diagnostic patterns.

### 3.3 Association between baseline UV damage and therapeutic improvement

**Figure 2:**
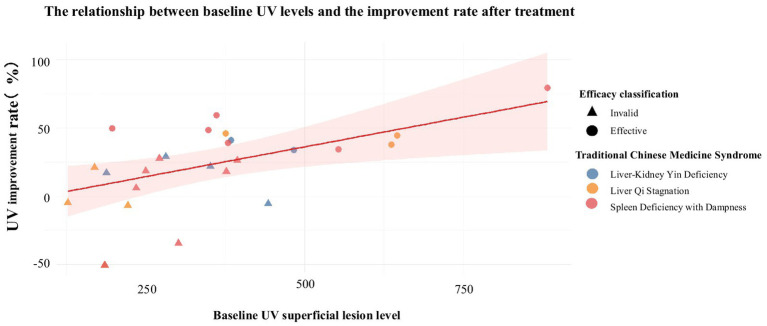
Association between baseline severity of superficial ultraviolet (UV) damage and post-treatment improvement rate, stratified by efficacy outcome and traditional Chinese medicine (TCM) syndrome.

Analysis of the relationship between baseline severity of UV-induced damage and subsequent improvement revealed a distinct pattern (Figure 2). A clear negative correlation was observed between the baseline level of superficial UV spots and the post-treatment UV improvement rate. Patients who ultimately were classified as “effective” (green squares) generally started with moderate baseline UV levels and achieved higher improvement rates. Conversely, many “ineffective” patients (red circles) presented with either very high or very low baseline UV damage, suggesting potential ceiling effects or different pathophysiological substrates that are less responsive to the intervention. Notably, this relationship was modulated by the TCM syndrome. For instance, within comparable ranges of baseline UV damage, patients with spleen deficiency with dampness (triangles) tended to cluster in regions indicating better improvement, whereas those with Qi stagnation and blood stasis (inverted triangles) were consistently found in the low-improvement region, further emphasizing the interplay between baseline biophysical features and systemic syndrome diagnosis in determining treatment outcome.

Quantitative analysis of baseline multi-dimensional imaging revealed distinct phenotypic profiles associated with each TCM syndrome (Supplementary Figures S1–S4). At baseline, the liver–kidney Yin deficiency group presented with the most severe level of brown spots (pigmentation) compared to other syndromes (Supplementary Figure S1), objectively supporting the TCM theory linking Yin deficiency to hyperpigmentation tendencies. The spleen deficiency with dampness group showed a trend toward higher baseline levels of superficial UV lesions (Supplementary Figure S2). No statistically significant differences were found among syndromes for baseline red area (vascularity) levels (Supplementary Figure S3). These findings suggest that TCM syndromes correspond to specific, measurable differences in pre-treatment skin biophysical properties.

### 3.4 Multivariate phenotypic profiling and feature contributions

Principal component analysis (PCA) was used to delineate the underlying multivariate phenotypic structure among the four TCM syndrome groups based on quantified skin imaging features. The first two principal components (PCs) collectively explained 58.9% of the total variance (PC1: 40.6%, PC2: 18.3%). PC1, which captured the largest variance fraction, was primarily driven by UV spot metrics (both superficial and deep), with loadings of approximately 0.60 and 0.50, respectively. Therefore, PC1 can be biologically interpreted as a cumulative actinic damage axis—representing the overall burden of chronic ultraviolet exposure. PC2 was dominated almost entirely by brown spots (pigmentation), with a loading of approximately 0.95, establishing PC2 as a dyspigmentation axis that is largely independent of UV spot burden.

**Figure 3:**
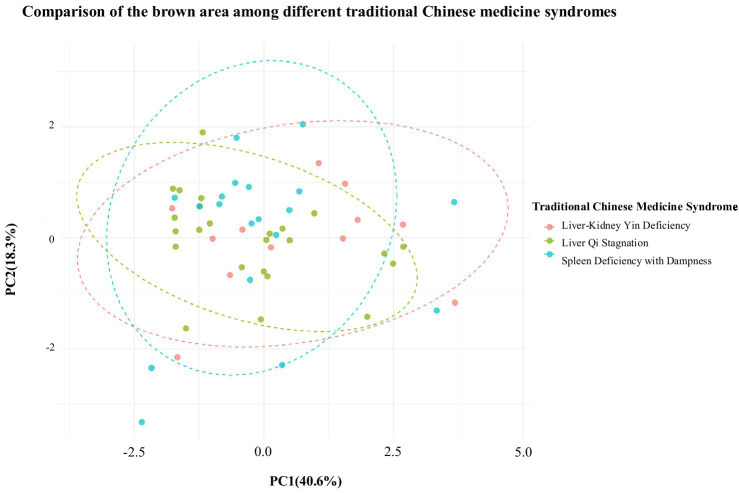
Mean principal component scores for each traditional Chinese medicine (TCM) syndrome group.

The separation of the liver–kidney Yin deficiency group along the positive direction of PC2 (Figure 3), therefore, carries a specific biological meaning: This syndrome is associated with a phenotype characterized by disproportionate pigmentation relative to the degree of actinic damage. In TCM theory, Yin deficiency is believed to generate endogenous “deficiency-fire” (虚火), which in dermatological terms may correspond to upregulated melanogenesis or impaired pigment clearance. This objective finding provides the first direct imaging-based evidence linking this classical TCM syndrome to a distinct biophysical signature. PC1 (40.6% variance) is primarily driven by UV spots (loadings >0.5), thereby representing cumulative actinic damage. PC2 (18.3%) is dominated by brown spots (loading ≈0.95), representing dyspigmentation. The separation of the liver–kidney Yin deficiency group along PC2 suggests that this syndrome is specifically associated with pigmentation biology, not merely UV damage.

The corresponding loading plot (Supplementary Figure S5) reveals the contribution of individual skin indicators to these PCs. Brown spots (pigmentation) exhibited a very high positive loading on PC2 (loading ≈ 0.95), indicating that this dimension primarily captures variability in dyspigmentation. This strongly aligns with the distinct clustering of the liver–kidney Yin deficiency group along PC2, objectively corroborating its association with pronounced pigmentation. Conversely, UV spots (superficial) and UV spots (deep) exhibited high positive loadings on PC1 (loadings ≈ 0.60 and 0.50, respectively), establishing PC1 as a dimension representing the severity of actinic damage. Red areas (vascularity) contributed moderately to both PCs. This decomposition confirms that the major axes of phenotypic variation in photoaging are primarily driven by separable components of pigmentation and direct UV damage and that TCM syndromes are associated with distinct positions within this objective phenotypic landscape.

### 3.5 Intercorrelations among skin imaging features and with treatment response

The correlational structure among baseline skin imaging parameters and their relationship to therapeutic outcome was further elucidated (Supplementary Figure S6; Supplementary Table S2). Analysis revealed a significant negative correlation (r = −0.32, *p* < 0.05) between the baseline severity of the brown spots (pigmentation) and the subsequent UV improvement rate following treatment. This suggests that patients with more pronounced baseline pigmentation experienced less improvement in UV-induced damage metrics. In contrast, the baseline level of UV superficial lesions showed no significant correlation with its own improvement rate (r = −0.12, *p* > 0.05). The red zone (vascularity) at baseline was weakly positively correlated with the UV improvement rate (r = 0.29).

**Figure 4:**
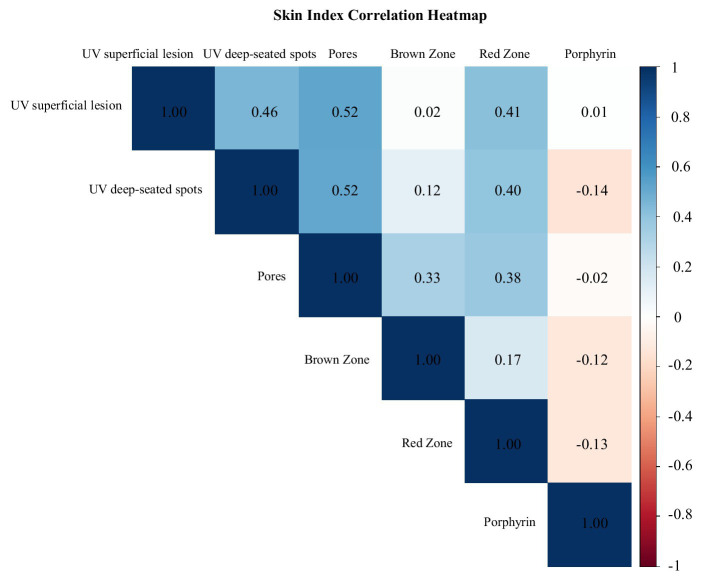
Intercorrelation heatmap of baseline multi-dimensional skin imaging features.

Furthermore, analysis of inter-feature correlations at baseline (Figure 4) delineated the intrinsic structure of the photodamage phenotype. A strong positive correlation was observed between UV superficial lesions and UV deep-seated spots (r = 0.52), indicating that these measures often covary as markers of actinic damage. Pores demonstrated a moderate positive correlation with the red zone (r = 0.62). Interestingly, porphyrin levels (reflecting sebaceous activity and *Cutibacterium acnes* presence) showed minimal to weak correlations with other structural and pigmentary features, suggesting that it represents a relatively independent physiological dimension within the photoaging profile.

**Figure 5:**
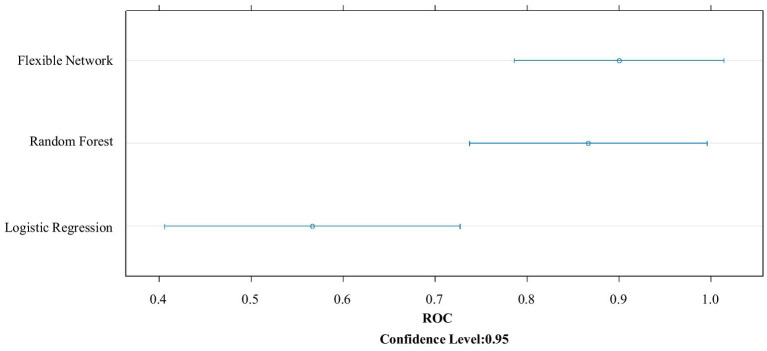
Comparative performance of three machine learning models in predicting treatment efficacy.

These correlation patterns reinforce the multi-dimensional nature of photographs captured by the imaging platform and provide a rationale for the distinct feature loadings observed in the principal component analysis. They also highlight specific baseline characteristics, particularly pigmentation severity, that may influence the therapeutic response trajectory (Figure 5).

### 3.6 Integrative predictive model achieves high accuracy

**Figure 6:**
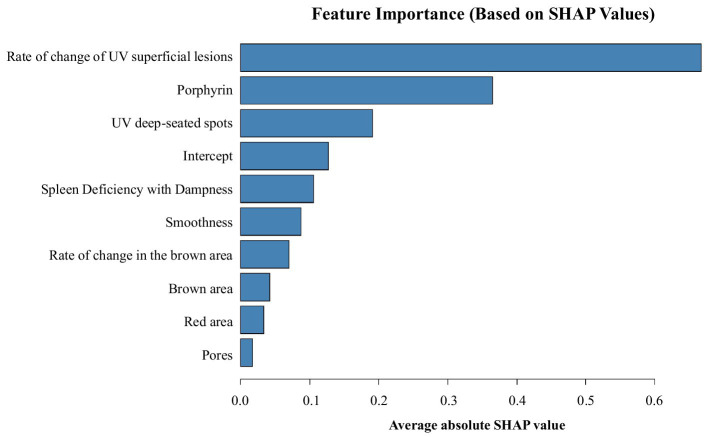
Feature importance ranking based on mean absolute SHAP (Shapley Additive exPlanations) values from the final predictive model.

To interpret the high-performing XGBoost model and identify the key drivers of treatment outcome, Shapley Additive Explanations (SHAP) analysis was performed (Figure 6; Supplementary Figure S8). Top predictors and their biological/clinical meaning are summarized below. The “rate of change of UV superficial lesions” emerged as the most influential feature, exhibiting the highest mean absolute SHAP value. This finding carries important clinical implications: Dynamic treatment response—that is, how quickly UV spots improve during therapy—is a stronger predictor of final outcome than any single baseline measurement. Clinically, this suggests that early assessment of UV spot changes (e.g., at week 4 or 6) could serve as a real-time treatment guide, allowing clinicians to adjust interventions before the 12-week endpoint. Porphyrin intensity (reflecting sebaceous activity and *Cutibacterium acnes* colonization) ranked as the second most important predictor. This novel finding suggests that the skin microbiome or sebaceous gland activity may modulate treatment response in photography, a hypothesis that has received little attention in the dermatology literature.

Notably, the TCM syndrome “spleen deficiency with dampness” was ranked as the fifth most important feature overall and was the highest-ranking non-imaging variable. Its SHAP value placed it ahead of several quantified baseline imaging metrics, including skin smoothness, brown spots, red areas, and pores. This provides model-based evidence that TCM syndrome classification contributes unique predictive information that cannot be fully captured by objective biophysical measurements alone. In other words, the holistic, systemic assessment embodied by TCM diagnosis complements localized skin imaging, reinforcing the value of integrative approaches in precision dermatology.

The SHAP analysis reveals a three-layered hierarchy of predictors: (1) dynamic treatment response (UV change rate), (2) baseline physiology (porphyrin and UV spots), and (3) systemic TCM diagnosis (spleen deficiency with dampness). Each layer contributes independently to predicting therapeutic outcomes, suggesting that optimal prediction requires integrating temporal, biophysical, and constitutional factors.

### 3.7 Interpretability of the predictive model: key determinants of treatment response

To interpret the high-performing XGBoost model and identify the key drivers of treatment outcome, Shapley Additive Explanations (SHAP) analysis was performed (Figure 6; Supplementary Figure S8). The rate of change of UV superficial lesions emerged as the most influential feature, exhibiting the highest mean absolute SHAP value. This was followed by porphyrin intensity and UV deep-seated spots, indicating that dynamic changes in actinic damage and baseline sebaceous activity were critical predictors. Notably, the TCM syndrome “spleen deficiency with dampness” was ranked as the fifth most important feature overall and was the highest-ranking non-imaging variable. Its SHAP value placed it ahead of several quantified baseline imaging metrics, including skin smoothness, brown spots, red areas, and pores. This result provides model-based evidence that the TCM syndrome classification contributes substantial, unique predictive information that complements and extends the data provided by objective biophysical measurements alone. The analysis thus elucidates the specific features—spanning dynamic treatment response, baseline physiology, and holistic TCM diagnosis—that collectively underpin the model’s ability to stratify patients by likely therapeutic outcome.

## 4 Discussion

This study provides compelling evidence that the holistic diagnostic categories of TCM are associated with distinct, quantifiable multi-dimensional phenotypes in facial photoaging, as captured by modern non-invasive imaging. This finding bridges a critical gap between traditional diagnostic theory and contemporary biophysical measurement, offering a novel framework for the objective characterization of TCM syndromes (19). Specifically, the strong association between the “*spleen deficiency with dampness*” syndrome and prominent superficial UV spots presents a novel, objective biophysical correlate. In TCM theory, the spleen governs transportation and transformation; its deficiency with dampness accumulation often manifests as tissue edema, poor nourishment, and impaired defensive *Qi* (20). Biophysically, this may translate to compromised skin barrier function, altered local inflammatory responses to UV exposure, or differences in water content affecting light scattering properties—all of which could influence the appearance and quantification of superficial photodamage (21). This correlation provides a testable hypothesis for future pathophysiological investigations.

Similarly, the association between “Liver-Kidney Yin Deficiency” and increased parameters of brown spots aligns with TCM pathophysiology, where Yin deficiency leads to endogenous “deficiency-fire,” a state often correlated with hyperpigmentation disorders in clinical practice (22). Modern dermatology recognizes that chronic UV exposure disrupts melanogenesis regulation through complex signaling pathways involving oxidative stress and inflammation (23). Our findings suggest that the TCM syndrome of Yin deficiency may identify a subset of patients with a distinct biological predisposition toward dysregulated pigmentation following photodamage, potentially associated with specific inflammatory or metabolic profiles (24).

The exceptional performance (AUC = 0.99) of our integrative predictive model is a key finding. It underscores a crucial principle: while quantitative imaging provides powerful, high-dimensional objective data, the TCM syndrome label contributes unique, complementary information that significantly enhances predictive power (25). This suggests that TCM diagnosis encapsulates a holistic, integrative assessment of the patient’s pathophysiological state, encompassing systemic tendencies and constitutional factors that are not fully reducible to a set of isolated, localized skin measurements (26). The model’s performance advocates for a synergistic approach in precision dermatology, where conventional biomarkers are enriched with pattern-based diagnostic information. The SHAP analysis further confirmed the “spleen deficiency with dampness” syndrome as a top predictor, robustly validating its clinical relevance in forecasting outcomes for photography interventions and highlighting its central role in the integrative pathophysiology of skin aging from a TCM perspective (27).

### 4.1 Limitations and future directions

Our study has several limitations that should be considered when interpreting the results and that chart a course for future research. First, this was a single-center study with a specific demographic (Fitzpatrick skin types III–IV). Although the sample size (n = 60) was sufficient for proof-of-concept modeling, it remained modest, resulting in small numbers for some syndrome subgroups (e.g., Qi stagnation and blood stasis, n = 2, which were excluded from the primary analyses). This limits the stability of estimates for less prevalent syndromes and reduces generalizability to broader populations (28). Second, the predictive model was built and validated based on a specific standardized treatment protocol. Its generalizability to other treatment modalities (e.g., different laser parameters, topical regimens, or combination therapies) requires external validation (29). The remarkably high AUC, while encouraging, should be interpreted with caution in this context and confirmed in larger, independent cohorts. Third, we acknowledge that future studies should include male participants to assess the universality of these findings, which would be very valuable.

Future research should prioritize multi-center studies with larger, more diverse cohorts to externally validate the imaging phenotypes and the predictive model, thereby strengthening the evidence for robustness and transportability of our findings (29). Translational research linking these distinct imaging phenotypes to underlying molecular pathways, including a comparative analysis of local and systemic markers of inflammation, oxidative stress, extracellular matrix remodeling, and melanogenesis, is essential to deepen the biological understanding of what TCM syndromes represent in modern pathological terms (30). Finally, prospective intervention studies stratified by TCM syndrome could directly test the clinical utility of this integrative classification system for guiding personalized treatment selection, thereby moving from correlation to causation and realizing the promise of stratified medicine in dermatology (31, 32).

## 5 Conclusion

This proof-of-concept study provides empirical evidence that TCM syndromes correspond to specific, objective, multi-dimensional skin phenotype patterns. An integrative model combining TCM diagnosis and quantitative imaging biomarkers can predict therapeutic outcomes with high accuracy in this exploratory cohort. These findings bridge TCM theory and modern biophysical assessment, paving the way for data-driven, personalized dermatology, pending external validation.

## Data Availability

The original contributions presented in the study are included in the article/Supplementary material, further inquiries can be directed to the corresponding author.
