# Correction: Phenylbutazone concentrations in synovial fluid following administration via intravenous regional limb perfusion in the forelimbs of six adult horses

**DOI:** 10.3389/fvets.2026.1869864

**Published:** 2026-07-01

**Authors:** Molly O'Brien, Jonathan P. Mochel, Kevin Kersh, Chong Wang, Jarrod Troy

**Affiliations:** 1 Department of Veterinary Clinical Sciences, Iowa State University College of Veterinary Medicine, Ames, IA, United States; 2 Department of Biomedical Sciences, Iowa State University College of Veterinary Medicine, Ames, IA, United States; 3 Department of Veterinary Diagnostic and Production Animal Medicine, Iowa State University College of Veterinary Medicine, Ames, IA, United States; 4 College of Veterinary Medicine, University of Georgia, Athens, GA, United States; 5 Department of Statistics, Iowa State University, Ames, IA, United States

**Keywords:** phenylbutazone, perfusion, horse, NSAID, anti-inflammatory, equine, IVRLP

There was a mistake in Tables 1, 2 as published. λ_z_ is mislabeled as “kel”, AUMC units are listed as μg·h/mL: the correct unit is μg·h^2^/mL, CL and Vd should be reported as CL/F and *V*_z_/*F*. In addition, there was a mistake in the caption of Table 1 & Table 2 as published. λ_z_ is mislabeled as “kel” and CL and Vd should be reported as CL/F and Vz/F. The corrected Tables 1, 2 and its captions appears below.

**Table 1:** Serum and radiocarpal joint synovial fluid non-compartmental pharmacokinetic analysis parameters (median [interquartile range]) for phenylbutazone (PBZ) following a single PBZ intravenous regional limb perfusion (IVRLP) using a 2.2 mg/kg PBZ dose in six equine forelimbs.

Parameter	Serum PBZ	Synovial PBZ
AUC_inf_ (μg^*^h/mL)	106.2 [98–118.5]	43.6 [30.3–53.2]
AUC_last_ (μg^*^h/mL)	101.4 [95.3–113.5]	42.3 [28.9–47]
AUMC_inf_ (μg^*^h^2^/mL)	829 [689.3–984.8]	360.3 [249.7–376.3]
CL/F (mL^*^h^−1^/kg)	20.7 [18.6–22.5]	
Cmax (μg/mL)	14.8 [12.7–15.4]	3.9 [3.3–5.4]
*T*max (h)	0.5 [0.5–0.5]	2.3 [1.5–3]
*T*_1/2_ (h)	4.7 [4.5–5.4]	4.5 [4–5.7]
λ_z_ (h^−1^)	0.15 [0.13–0.15]	0.16 [0.12–0.18]
MRT_inf_ (h)	7.7 [7–8.5]	8.3 [6.4–12.2]
*V*_z_/*F* (mL/kg)	139.2 [132.4–170.7]	

All data was generated using non-compartmental analysis. AUC, area under the curve; AUMC, area under the moment curve; CL/F, clearance rate; Cmax, maximum concentration; Tmax, time until Cmax; *T*_1/2_, terminal half-life; λ_z_, terminal disposition rate constant; MRT, mean residence time; *V*_z_/*F*, volume of distribution.

**Table 2:** Serum and radiocarpal joint synovial fluid non-compartmental pharmacokinetic analysis parameters [median (interquartile range)] for oxyphenbutazone (OBZ) following a single PBZ intravenous regional limb perfusion (IVRLP) using a 2.2 mg/kg PBZ dose in six equine forelimbs.

Parameter	Serum OBZ	Synovial OBZ
AUC_inf_ (μg^*^h/mL)	24.4 [17.3–30.4]	10.4 [7.9–15.7]
AUC_last_ (μg^*^h/mL)	18.6 [12.5–23.8]	7.6 [5.3–10.9]
AUMC_inf_ (μg^*^h^2^/mL)	429.2 [220.5–511.5	203.7 [115.5–235.2]
Cmax (μg/mL)	1.4 [1.3–1.6]	0.6 [0.37–0.63]
Tmax (h)	5 [5]	5 [5]
T_1/2_ (h)	8.3 [7.7–9.2]	9.2 [6.9–12.6]
λ_z_ (h^−1^)	0.09 [0.08–0.09]	0.08 [0.06–0.1]
MRT_inf_ (h)	14.8 [12.8–16.8]	17 [13.4–22.1]

All data was generated using non-compartmental analysis. AUC, area under the curve; AUMC, area under the moment curve; CL/F, clearance rate; Cmax, maximum concentration; Tmax, time until Cmax; *T*_1/2_, terminal half-life; λ_z_, terminal disposition rate constant; MRT, mean residence time; *V*_z_/*F*, volume of distribution.

λ_z_ is mislabeled as “kel,” CL and Vd should be reported as CL/F and *V*_z_/*F* throughout the **Methods** and **Discussion**.

A correction has been made to **Methods**, *2.8 Pharmacokinetic analysis*, Paragraph 1:

Pharmacokinetic analysis of total PBZ and OBZ within serum and synovial fluid concentrations was performed using a statistical moment (i.e., non-compartmental) approach in commercial software (PKanalix, MonolixSuite 2020R1, Lixoft, France). Time versus concentration figures for PBZ and OBZ were produced via a commercial program (GraphPad Prism 8.0, Graphpad Software, Inc., La Jolla, CA, USA). Standard PK parameters were generated for individual horses as follows:

- Maximum PBZ and OBZ concentration, Cmax;
- Time of maximum PBZ and OBZ concentration, Tmax;
- Area under PBZ and OBZ concentration-time curve, AUC_last_;
- Area under the moment curve, AUMC_inf_;
- PBZ and OBZ mean residence time, MRT = AUMC_inf_/AUC_inf_
- Slope of the elimination phase λ_z_, computed by linear regression of the logarithmic concentration vs. time curve during the elimination phase, λ_z_;
- PBZ and OBZ terminal half-life, *T*_1/2_(λ_z_) = ln(2)/λ_z_;
- PBZ apparent clearance, CL/F = Dose/AUC_inf_;
- Apparent volume of distribution of PBZ during the elimination phase, *V*_z_/*F* = Dose/(AUC_inf_ × λ_z_);

For data analysis, the first value below the LLOQ was inferred to be LLOQ/2, and subsequent data points were excluded from the analysis. A linear/log trapezoidal rule was used to estimate the area under PBZ and OBZ time curves. Summary statistics on the individual pharmacokinetic parameters were performed thereafter to derive the geometric mean, median, and (min–max) range.

A correction has been made to **Discussion**, paragraph 1:

The primary objective of this paper was to determine the concentrations and pharmacokinetics of PBZ, and its active metabolite OBZ, in equine serum and synovial fluid following a single administration PBZ-IVRLP administration in standing adult horses. The administration of PBZ-IVRLP in standing adult horses resulted in serum PBZ concentrations that were significantly higher than in RCJ synovial fluid in this study. This is in contrast to reported synovial and serum antibiotic concentrations following antibiotic-IVRLP administration in horses (8, 9, 16). In this study, serum PBZ clearance, terminal half-life, and volume of distribution (CL/*F*, *T*_1/2_, *V*_z_/*F*) were similar to those reported following systemic PBZ administration (11, 12). The Tmax and Cmax for serum PBZ may have been similar to systemic administration as well but no serum samples were taken during the IVRLP procedure which is a limitation of this study. These findings show that PBZ remained within the peripheral vasculature more than it diffused into the synovial fluid of the RCJ. The *T*_1/2_ for synovial PBZ concentrations was similar to systemic serum indicating similar release of PBZ from the RCJ into systemic serum. This would indicate that the dosing schedule would likely need twice a day administration which may not be clinically practical or that PBZ is not tissue soluble enough to diffuse into the RCJ within the short tourniquet time of 30 min (11, 12). Additionally, OBZ concentrations were similar to those previously reported following systemic PBZ administration (12). These findings demonstrate that a single PBZ-IVRLP, at 2.2 mg/kg), was similar to a single systemic intravenous administration of PBZ in healthy, standing adult horses with no additional clinical benefits.

The original version of this article has been updated.

