# Correction: Integrative analysis links traditional Chinese medicine syndrome differentiation to multi-dimensional skin phenotypes and predicts therapeutic response in photographs

**DOI:** 10.3389/fmed.2026.1895485

**Published:** 2026-07-03

**Authors:** Zhili Dou, Pingmei Shi, Juan Tan, Rong Jing, Caixia Hui, Yaoxia Zhang, Ruixi Li, Yuehao Sun, Yunlei Liu

**Affiliations:** 1 School of Basic Medical Sciences, Yan‘an University, Yan'an, China; 2 The Affiliated Hospital of Yan'an University, Yan'an, China; 3 The Department of Traditional Chinese Medicine of the Affiliated Hospital of Yan'an University, Yan'an, China; 4 Rehabilitation Medicine Department of Affiliated Hospital of Yan'an University, Yan'an, China; 5 Department of Chemical Engineering, Yan‘an University, Yan'an, China

**Keywords:** machine learning, predictive modeling, skin imaging, syndrome differentiation, traditional Chinese medicine

Affiliation “The Affiliated Hospital of Yan'an University, Yan'an, China” was omitted for author Pingmei Shi. This affiliation has now been added for author Pingmei Shi.

Affiliation “The Department of Traditional Chinese Medicine of the Affiliated Hospital of Yan'an University, Yan'an, China” was removed for author Pingmei Shi.

Author Rong Jing should have the affiliation “Rehabilitation Medicine Department of Affiliated Hospital of Yan'an University, Yan'an, China” instead of “The Department of Traditional Chinese Medicine of the Affiliated Hospital of Yan'an University, Yan'an, China”

Affiliation “Department of Chemical Engineering, Yan'an University, Yan'an, China” was omitted for author Yaoxia Zhang. This affiliation has now been added for author Yaoxia Zhang. Affiliation “Rehabilitation Medicine Department of Affiliated Hospital of Yan'an University, Yan'an, China” was removed for author Yaoxia Zhang.

The author list of the published paper was erroneously given as:

Zhili Dou^1^, Pingmei Shi^1, 2^, Juan Tan^2^, Rong Jing^2^, Caixia Hui^2^, Yaoxia Zhang^3^, Ruixi Li^2^, Yuehao Sun^1^ and Yunlei Liu^2*^

The correct author list reads:

Zhili Dou^1^^**†**^, Pingmei Shi^1, 2^^**†**^, Juan Tan^3^, Rong Jing^4^, Caixia Hui^3^, Yaoxia Zhang^5^, Ruixi Li^3^, Yuehao Sun^1^ and Yunlei Liu^3*^

In the abstract, there was an error of the system mentioned in the sentence:

“Baseline multi-modal facial imaging was performed using the Visia^®^ CA system or equivalent, quantifying features across four dimensions.”

This has been corrected to read:

“Baseline multi-modal facial imaging was performed using the CBS system or equivalent, quantifying features across four dimensions.”

There was an error in system mentioned in section **1 Introduction**, paragraph 1:

“Meanwhile, non-invasive imaging systems (e.g., Visia^®^) can objectively quantify photoaging features such as pigmentation, vascularity, and texture (5, 6).”

A correction has been made to: “Meanwhile, non-invasive imaging systems (e.g., CBS) can objectively quantify photoaging features such as pigmentation, vascularity, and texture (5, 6). ”

There was an error in the dates and approval number in section **2 Materials and methods** *2.1 Study design and participants*, paragraph 1:

“This prospective, single-center, observational cohort study was conducted between 2025.02 to 2025.03 and was approved by the Institutional Review Board of the Affiliated Hospital of Yan'an University (Approval No: CXY-2021-122).”

A correction has been made to: “This prospective, single-center, observational cohort study was conducted between 2025.08 and 2025.11 and was approved by the Institutional Review Board of the Affiliated Hospital of Yan'an University (Approval No: CXY2021122).”

Author Pingmei Shi was erroneously omitted as equal contributing author. “These authors have contributed equally to this work and share first authorship” has been added for authors Zhili Dou and Pingmei Shi.

The Ethics Committee name in the **Ethics statement** was incorrect. It previously read:

“The studies involving humans were approved by Yan'an University Ethics Committee. The studies were conducted in accordance with the local legislation and institutional requirements. The participants provided their written informed consent to participate in this study. Written informed consent was obtained from the individual(s) for the publication of any potentially identifiable images or data included in this article.”

The correct **Ethics statement** is:

“The studies involving humans were approved by Affiliated Hospital of Yan'an University. The studies were conducted in accordance with the local legislation and institutional requirements. The participants provided their written informed consent to participate in this study. Written informed consent was obtained from the individual(s) for the publication of any potentially identifiable images or data included in this article.”

The original version of this article has been updated.

