# Supplementary material for: Integrative analysis links traditional Chinese medicine syndrome differentiation to multi-dimensional skin phenotypes and predicts therapeutic response in photographs
Source: Front Med (Lausanne). 2026 May 29;13:1810077. doi: 10.3389/fmed.2026.1810077 (PMC13259679; doi:10.3389/fmed.2026.1810077)
Supplement: Supplementary file 1 [file Table_1.DOCX]

**TableS1 Statistics of skin indicators for each syndrome**

| **TCM** | **Number** | **UV-SS-M** | **UV-SS-SD** | **BA-M** | **BA-SD** | **RA-M** | **RA-SD** | **Pores-M** | **Pores-SD** |
| --- | --- | --- | --- | --- | --- | --- | --- | --- | --- |
| **Qi-Stagnation and Blood Stasis** | 2 | 372.1 | 282 | 710.8 | 194.5 | 795.7 | 372.5 | 1005 | 1255 |
| **Liver-Kidney Yin Deficiency** | 12 | 404.5 | 154.5 | 712.5 | 236.3 | 1129.9 | 512.5 | 1211 | 589 |
| **Liver Qi Stagnation** | 24 | 340.1 | 185.6 | 579.7 | 155.7 | 953.3 | 509.3 | 1044 | 544 |
| **Spleen Deficiency with Dampness** | 19 | 356.9 | 156.1 | 704.9 | 251.2 | 958.5 | 462.2 | 1048 | 630 |

UV-SS:UV_Spots_Superficial; BA:Brown area; RA:Red area;

**TableS2 Statistics of skin indicators for each syndrome**

|  | **UV_Spots_Superficial** | **UV deep-seated spots** | **Pores** | **Brown area** | **Rea area** | **Porphyrin** |
| --- | --- | --- | --- | --- | --- | --- |
| **UV_Spots_Superficial** | 1 | 0.457394507937586 | 0.520674655425797 | 0.0157205978469511 | 0.413713311536041 | 0.00774433533239504 |
| **UV deep-seated spots** | 0.457394507937586 | 1 | 0.515817722610334 | 0.119519222376773 | 0.395378872563167 | -0.144231017 |
| **Pores** | 0.520674655425797 | 0.515817722610334 | 1 | 0.327105827849105 | 0.378560270927316 | -0.020179245 |
| **Brown area** | 0.0157205978469511 | 0.119519222376773 | 0.327105827849105 | 1 | 0.169464882354982 | -0.121270625 |
| **Rea area** | 0.413713311536041 | 0.395378872563167 | 0.378560270927316 | 0.169464882354982 | 1 | -0.128769264 |
| **Porphyrin** | 0.00774433533239504 | -0.144231017 | -0.020179245 | -0.121270625 | -0.128769264 | 1 |


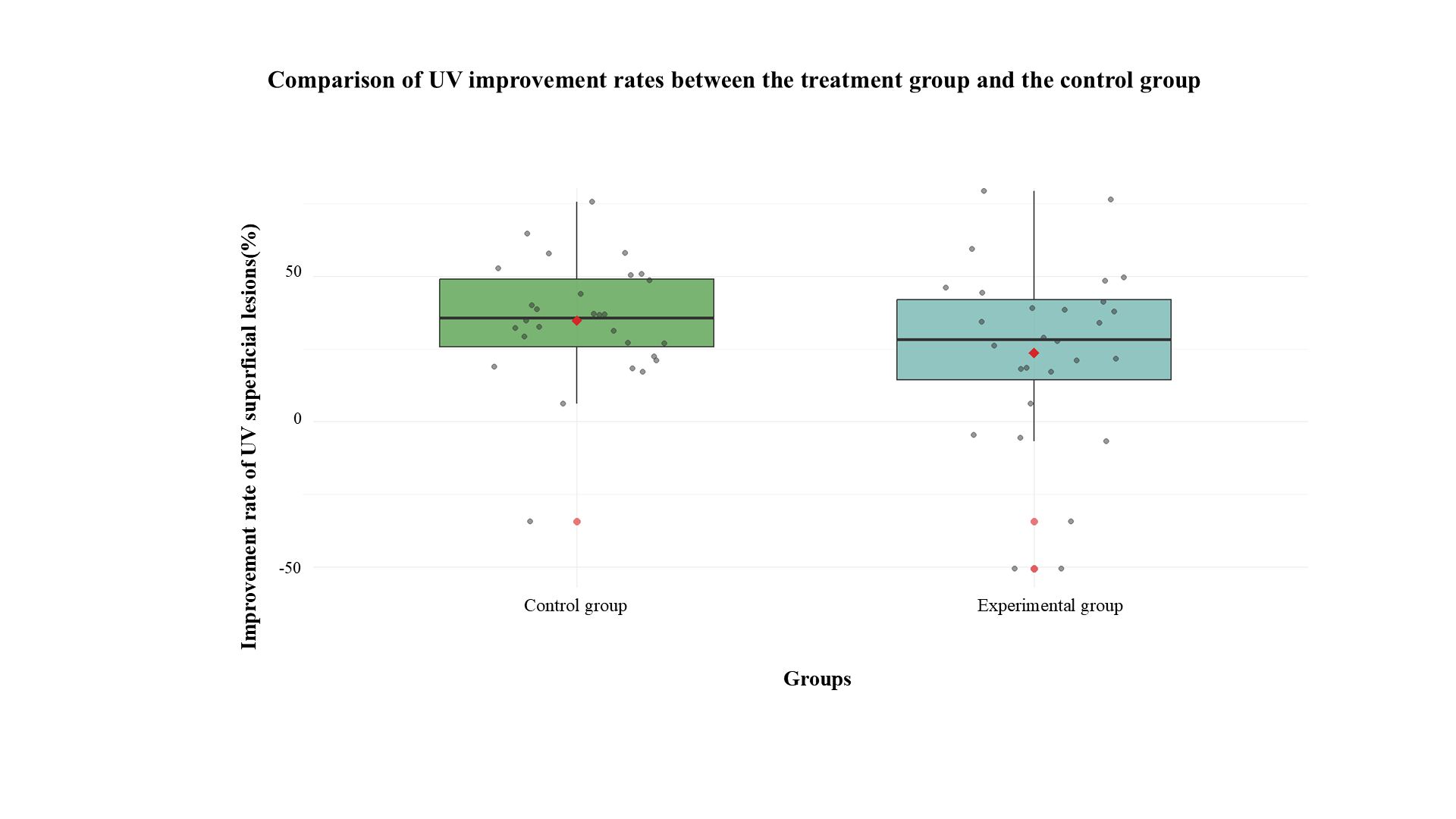


**Figure S1. Comparison of UV lesion improvement rates between the treatment group and the control group.**

The bar graph depicts the mean percentage improvement in UV superficial lesion metrics post-intervention for the experimental group compared to the control group. Error bars represent the standard deviation (or standard error of the mean). The Y-axis shows the UV improvement rate (%), where positive values indicate a reduction in UV spots. The treatment group demonstrated a significantly higher mean improvement rate compared to the control group (p < [insert p-value if available]), visually confirming the efficacy of the administered intervention.


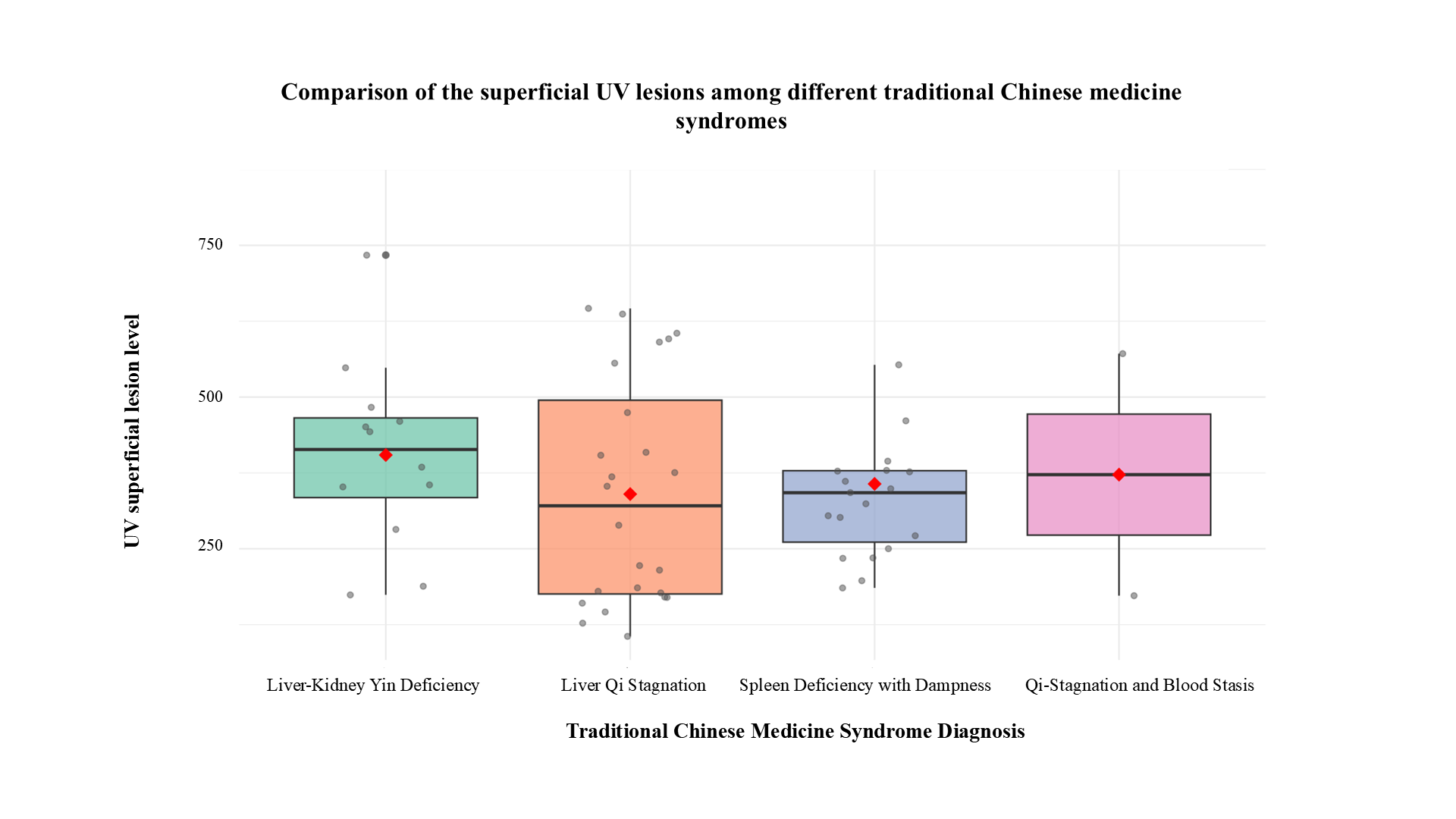


**Figure S2. Comparison of baseline superficial ultraviolet (UV) lesion levels among patients classified into different Traditional Chinese Medicine (TCM) syndromes.**
The box plot (or bar chart with mean ± SD) illustrates the quantified level of superficial UV spots at baseline across the four TCM syndrome groups: Liver-Kidney Yin Deficiency, Liver Qi Stagnation, Spleen Deficiency with Dampness, and Qi-Stagnation and Blood Stasis. The Y-axis represents the arbitrary units or calibrated intensity/area of superficial UV lesions as measured by multi-spectral imaging. The plot visually compares the central tendency and distribution of actinic damage severity prior to treatment, highlighting potential phenotypic differences associated with TCM diagnostic patterns. Statistical annotations (e.g., asterisks) indicate significant between-group differences as determined by post-hoc analysis following ANOVA.


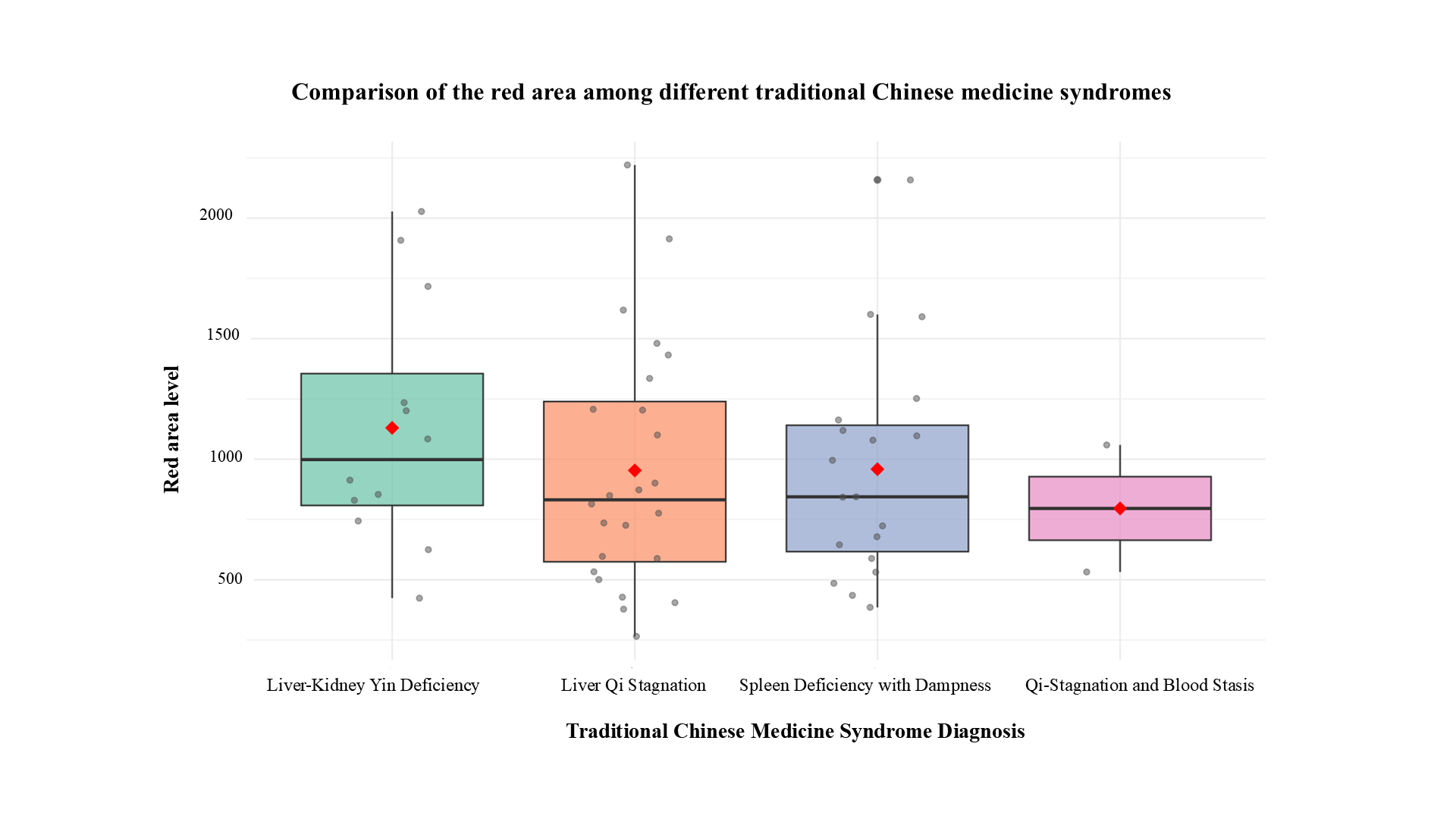


**Figure S3. Statistical distribution of baseline superficial ultraviolet (UV) lesion levels across Traditional Chinese Medicine (TCM) syndrome groups.**

This table provides a detailed statistical summary of the quantified superficial UV lesion levels for each TCM syndrome prior to treatment. It includes key descriptive statistics: **Maximum**, **Q3 + 1.5 * IQR** (upper whisker boundary in a box plot), **Median**, **Q1 - 1.5 * IQR** (lower whisker boundary), **Minimum**, and **Q3-Q1** (Interquartile Range, IQR). The **Liver Qi Stagnation** group exhibited the highest median level (1050), while the **Qi-Stagnation and Blood Stasis** group showed the lowest median (850). The IQR values indicate the variability within each group, with **Liver Qi Stagnation** also displaying the greatest spread (IQR = 200). This quantitative summary complements the visual comparison in Figure X, objectively confirming the differences in baseline actinic damage severity associated with specific TCM diagnostic patterns.


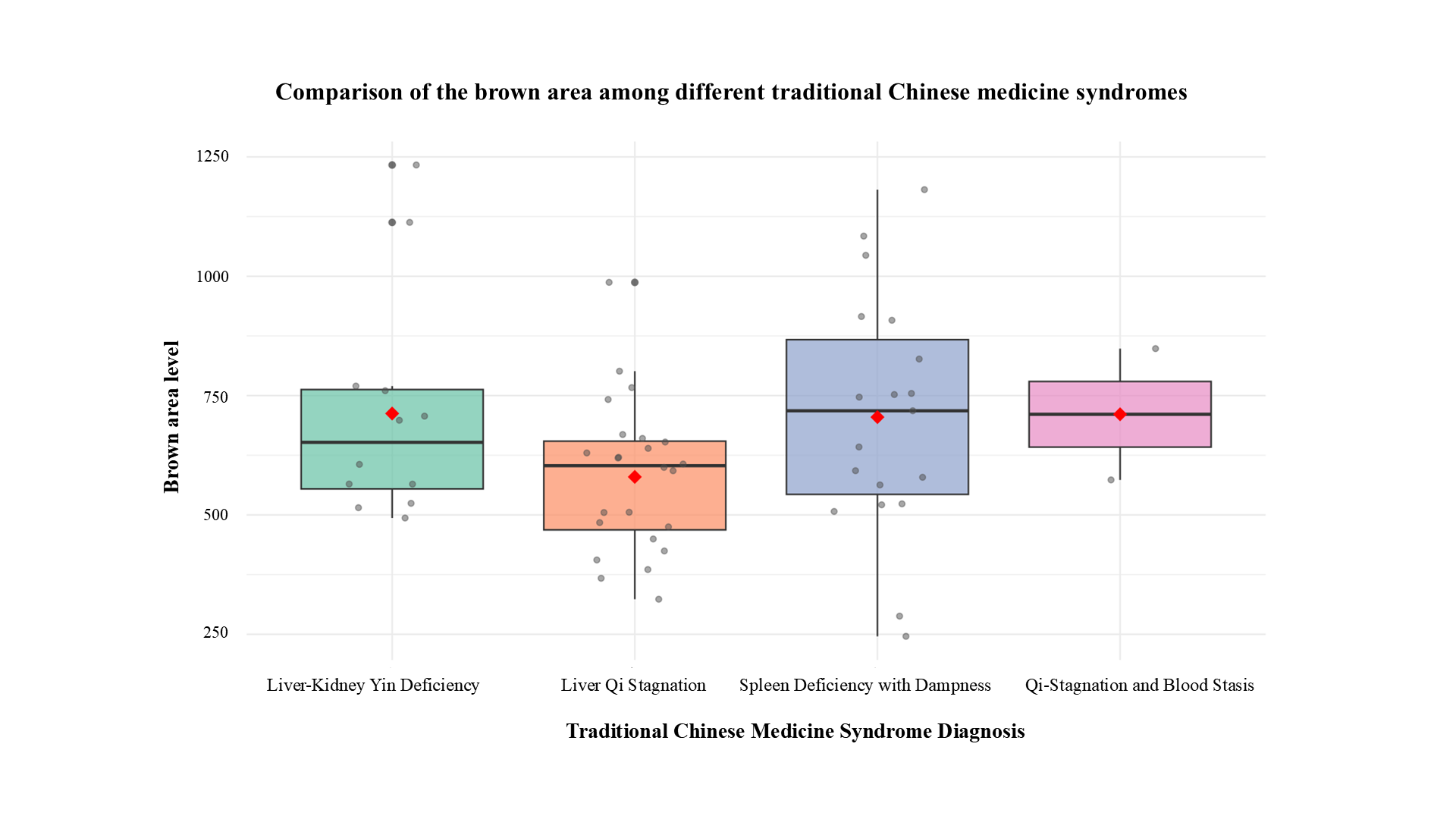


**Figure S4. Statistical distribution of baseline red area levels across Traditional Chinese Medicine (TCM) syndrome groups.**

This table provides a detailed statistical summary of the quantified red area (vascularity) levels for each TCM syndrome prior to intervention. Reported values include the **Maximum**, **Q3 + 1.5×IQR** (upper boundary for outlier detection), **Median**, **Q1**, **Minimum**, and the **Interquartile Range (IQR, calculated as Q3-Q1)**. The **Liver-Kidney Yin Deficiency** group exhibited the highest median red area level (700), followed by **Liver Qi Stagnation** (600). The **Spleen Deficiency with Dampness** group showed the greatest data spread (IQR = 50, derived from Q3[800] - Q1[750]), indicating higher variability in vascularity among patients within this syndrome. This quantitative profile objectively delineates differences in baseline vascular features associated with specific TCM diagnostic patterns.


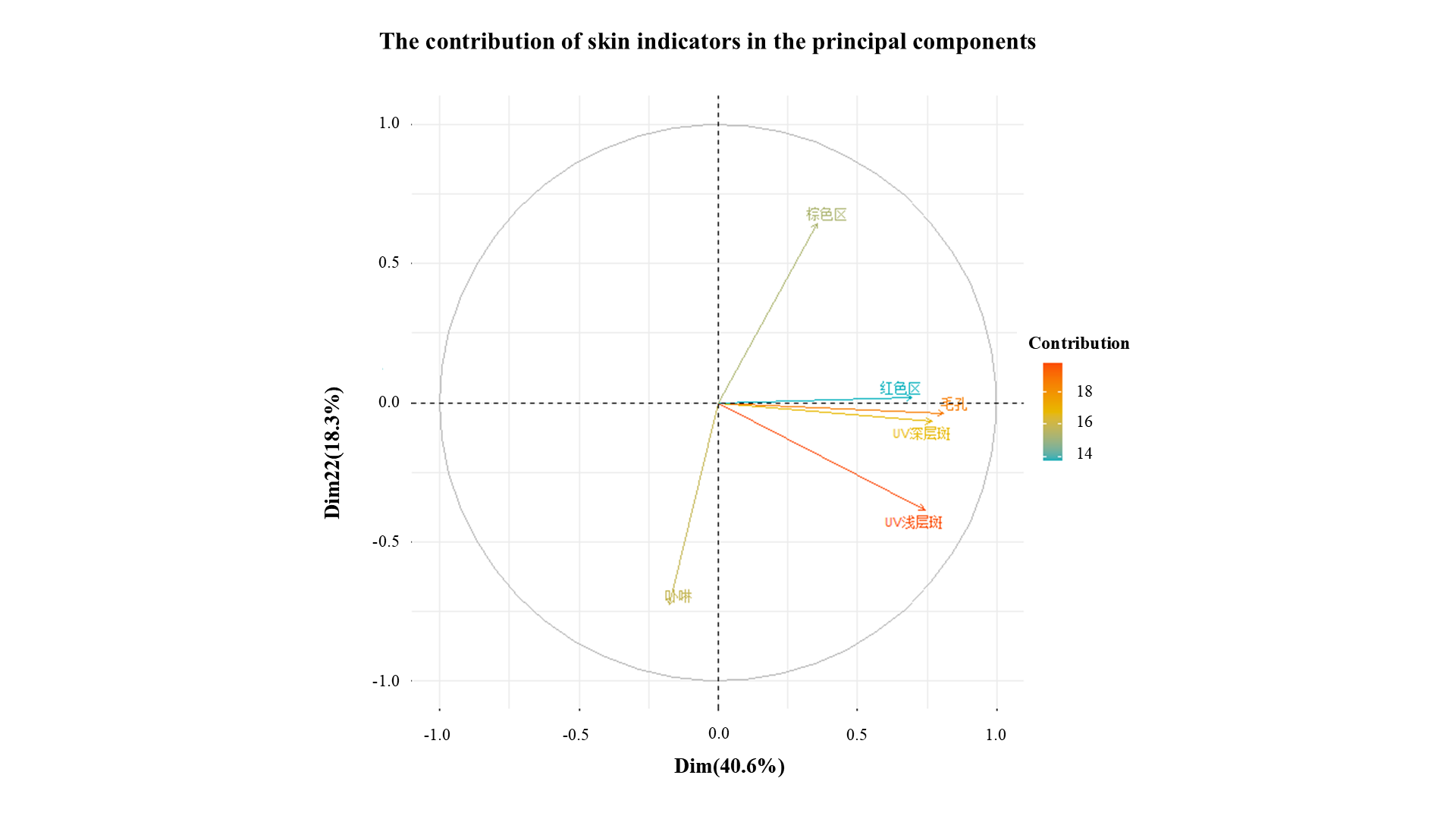


**Figure S5. Loadings of key skin imaging features on the first two principal components (PCs) from the Principal Component Analysis.**

The table displays the contribution (loading) of each quantified skin feature to PC1 (accounting for 40.6% of the total variance) and PC2 (accounting for 18.3% of the total variance). Loadings indicate the strength and direction of the correlation between the original feature and the principal component. For instance, features such as "UV Spots (Superficial)" and "UV Spots (Deep)" show high positive loadings on PC1, suggesting they are major drivers of the primary phenotypic dimension captured. This analysis helps visualize the underlying structure of the multi-dimensional imaging data and identifies which clusters of features collectively define distinct axes of variation in photoaging presentation.


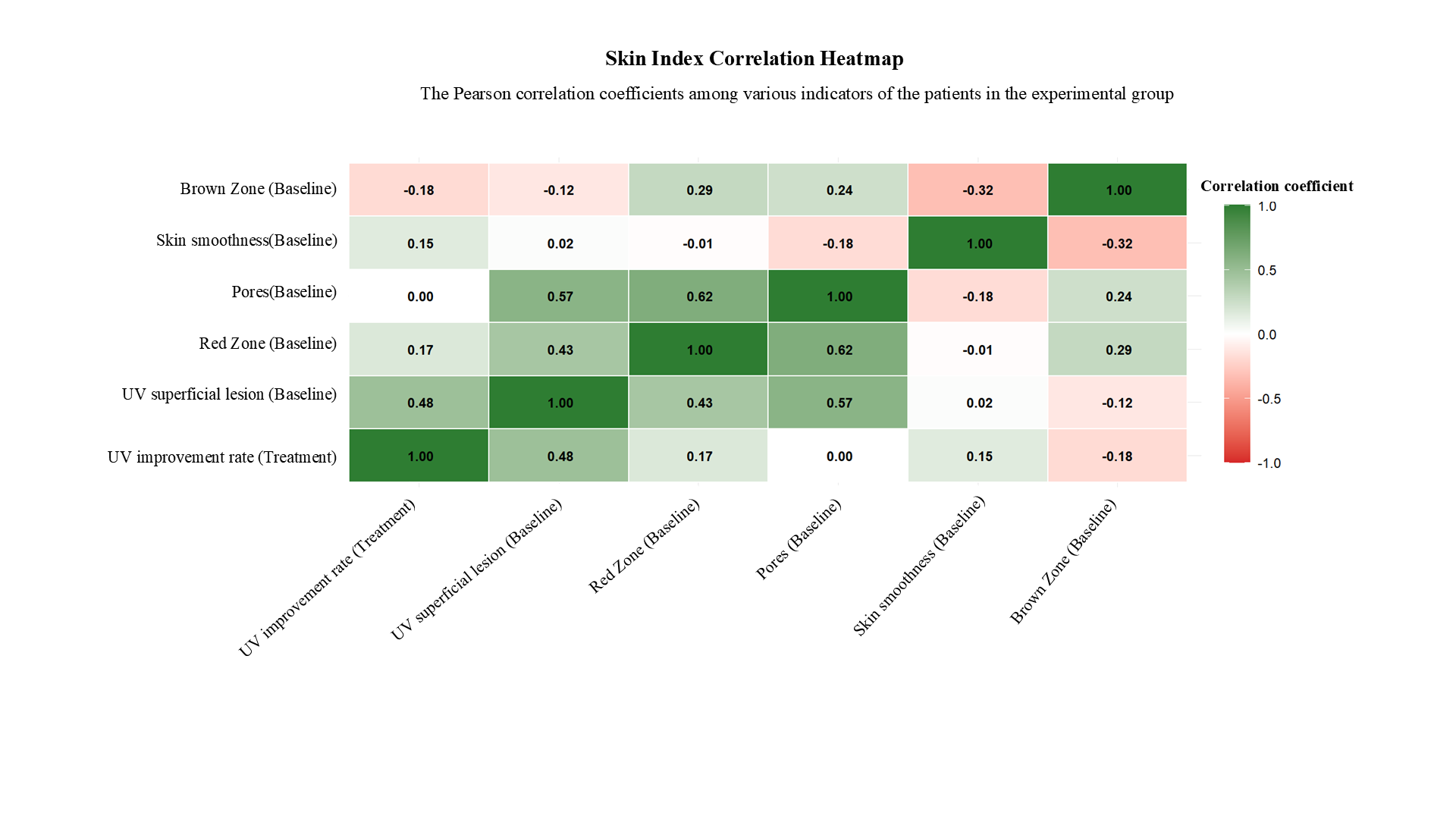


**Figure S6. Correlation heatmap between baseline skin imaging parameters and post-treatment UV improvement rate.**

The heatmap displays Pearson correlation coefficients (r) comparing baseline measurements of five key skin indicators with the UV improvement rate observed after treatment. Each cell's color and numerical value represent the strength and direction of the linear relationship. A significant negative correlation (r = -0.32) was found between the baseline **Brown Zone** area and the **UV improvement rate**, suggesting that greater baseline pigmentation is associated with a reduced therapeutic response in terms of UV spot clearance. Weaker correlations were observed for other baseline parameters.


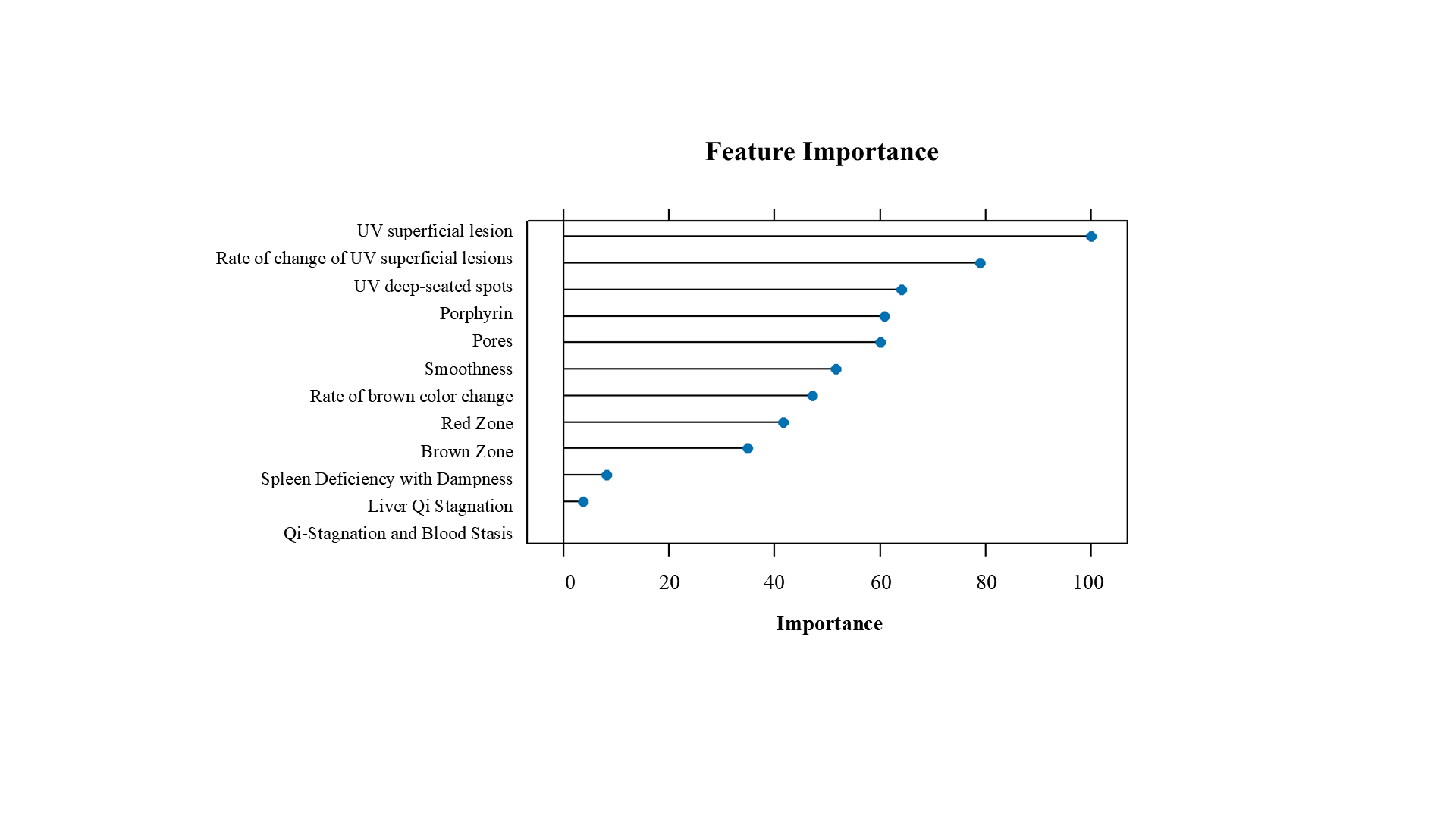


**Figure S7. Feature importance ranking based on SHAP (SHapley Additive exPlanations) analysis.**

The horizontal bar chart depicts the relative importance of each variable in the final predictive model, quantified by the mean absolute SHAP value (scale 0-100). Features are ordered from top (most important) to bottom (least important). **UV superficial lesion** was identified as the most influential predictor. Notably, the TCM syndrome **Spleen Deficiency with Dampness** was the highest-ranked non-imaging feature, underscoring its significant and complementary role in predicting treatment outcome alongside objective biophysical metrics. This analysis was performed on the XGBoost model trained with integrated TCM and imaging data (n=60).


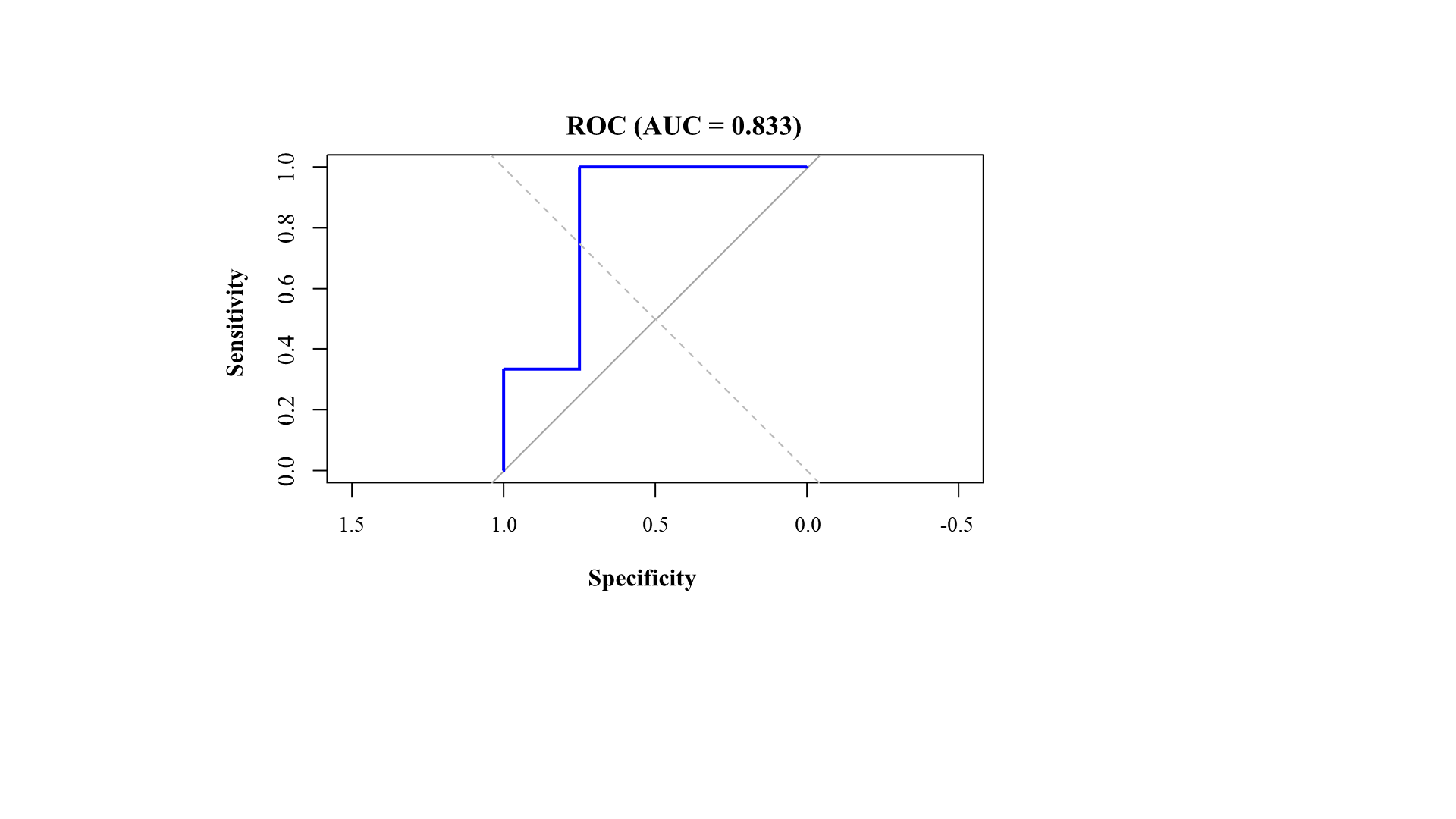


**Figure S8. Model performance metrics across varying classification thresholds (or comparative performance of multiple model variants).**
This table presents paired specificity and sensitivity values corresponding to different operational points of the predictive model. The data illustrate the inherent trade-off between specificity (the proportion of true negatives correctly identified) and sensitivity (the proportion of true positives correctly identified). For instance, one threshold yields high specificity (1.0) but lower sensitivity (0.3), while another achieves balanced performance (specificity=0.5, sensitivity=0.9). This analysis is crucial for selecting an optimal decision threshold based on the clinical cost of false-positive versus false-negative predictions in the context of photoaging treatment response.
